# Malnutrition matters: Association of stunting and underweight with early childhood development indicators in Nepal

**DOI:** 10.1111/mcn.13321

**Published:** 2022-01-20

**Authors:** Manisha L. Shrestha, Kelly E. Perry, Basant Thapa, Ramesh P. Adhikari, Amy Weissman

**Affiliations:** ^1^ FHI 360 Nepal Office Kathmandu Nepal; ^2^ FHI 360 Asia Pacific Regional Office Bangkok Thailand; ^3^ Helen Keller International Nepal Office Lalitpur Nepal

**Keywords:** early childhood development, malnutrition, Multiple Indicator Cluster Survey (MICS), Nepal, stunting, underweight, wasting

## Abstract

Malnutrition is a threat to optimal child development, with its occurrence during foetal and infancy stages associated with poor cognitive, motor and socio‐emotional skills. However, information on the effects of various types of malnutrition on early childhood development (ECD) is limited in Nepal. To assess the association of stunting, wasting and underweight (three prominent forms of malnutrition) with the four domains of the ECD index (literacy‐numeracy, physical, social‐emotional and learning development) among children 36–59 months of age, we conducted an adjusted logistic regression using Nepal's national household Multiple Indicator Cluster Survey (MICS) 2019 data set. The study sample consisted of children aged 36–59 months (*n* = 2871). Children were considered developmentally on track if they met criteria in each of the four ECD domains. Regarding ECD status of children 36–59 months old, 35% of children were not developmentally on track for the ECD index. The adjusted odds ratio indicated that stunting was associated with lower odds of not being developmentally on track according to the ECD index as well as the literacy‐numeracy, physical and learning domains of the ECD index. Likewise, underweight was associated with lower odds of not being developmentally on track according to the ECD index, primarily for ECD domains of literacy‐numeracy, physical and learning. Notably, no association between wasting and ECD indicators was observed. Children's nutrition status impacts child development outcomes. Adding ECD interventions, such as responsive and stimulating caregiving, within nutrition programmes among children who are stunted and underweight could improve child development outcomes.

## Key messages

- In Nepal, 35% of children in our data set did not display optimal early childhood development (ECD).
- Children who are malnourished, especially indicating signs of stunting and underweight, are likely to exhibit poor ECD indicators and may have limited cognitive capabilities in adulthood.
- Wasting demonstrated no notable association with ECD indicators.
- Study findings suggest that ECD interventions among children who are stunted and underweight could support optimal childhood development globally.
- Establishing partnerships between nutrition and ECD sectors to adopt the Nurturing Care Framework, especially targeting the first 5 years of life, would contribute to childhood development efforts.

## Graphical abstract

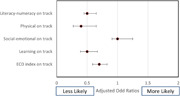

## 1 INTRODUCTION

Early child development (ECD) comprises physical, sensorimotor, social, emotional, language and cognitive aspects of growth (World Health Organization, 2021). The foetal stage period through 3 years of age are critical for growth, as 80% of total brain weight is formed by this age (World Health Organization et al., 2018). During the first few years after birth, children develop a set of age‐appropriate cognitive skills and social and emotional competencies which contribute to physical and mental wellbeing and economic productivity later in life (J. J. Heckman, 2007; J. Heckman et al., 2013; Hoddinott et al., 2008). Genes are the blueprint for brain development though a child's environment also plays a role (Centre on the Developing Child, 2021; UNICEF, 2014). An optimal environment for ECD during these fundamental years provides a foundation for good health and productivity throughout the life course (Lagercrantz, n.d). Thus, a child should receive nurturing care, including adequate health, nutrition, security and safety, opportunities for early learning and responsive care giving during early childhood to develop and thrive to their full potential in adulthood (McCormick et al., 2020; World Health Organization United Nations Children's Fund & World Bank Group, 2018). ECD is recognised as one of the most important predictors of future social capital and national productivity. However, the recent *Lancet* series on ECD (Black et al., 2017) reported that approximately 250 million children under five are at risk of not reaching their developmental potential (Chan et al., 2017).

Children's development can be compromised by a plethora of factors, including poverty, malnutrition, inadequate maternal nutrition, suboptimal breastfeeding, exposure to pollutants and toxic chemicals, HIV infection, mental health of caregivers, injuries, limited stimulation, neglect, maltreatment, disabilities and violence (Black et al., 2017). Nutrition—especially during foetal stage and infancy—provides a foundation for development of cognitive, motor, and socio‐emotional skills throughout childhood and adulthood (Prado & Dewey, 2014). Malnutrition has negative consequences for both the structure and functionality of the brain. Structurally, it causes tissue damage, growth retardation and reduced overall development of the human brain, leading to long‐term alterations in brain function resulting in permanent cognitive impairments (Atinc & Gustafsson‐Wright, 2013; Kar et al., 2008). Nutritional deficiencies including severe acute malnutrition, chronic undernutrition, and iron and iodine deficiency during pregnancy and infancy, are more likely to affect cognition, behaviour, and productivity throughout a child's school years and beyond (Prado & Dewey, 2014). In a study conducted in India, malnourished children were assessed on Gesell's developmental schedule from 4 to 52 weeks of age. Children with grades II and III malnutrition had poor development in all areas of behavior, including visual motor coordination, memory function compared to healthy counterparts (Upadhyay et al., 1989).

According to the 2020 Global Nutrition Report, stunting still affects 140.9 million children under the age of 5, and wasting affects 49.5 million children under 5. More than half (81.7 million) of stunted children reside in South Asia (World Health Organization, 2020). The region also holds the second highest number of children with low cognitive and socio‐emotional development test scores (Kang et al., 2018). While one trend analysis uncovered that stunting steadily declined in Nepal from 2001 to 2013, it has plateaued at above 30% since 2013, with wasting remaining inconsistent and high (Angela et al., 2020). In Nepal, recent Multiple Indicator Cluster Survey (MICS) data reveal that 31.5% of children under 5 years of age are stunted, 12% are wasted, and 24% are underweight (Government of Nepal National Planning Commission Central Bureau of Statistics & UNICEF Nepal, 2020). Only 12% of the poorest children are on track for adequate development of literacy numeracy skills (World Bank, 2020). A comparative study of MICS results from 30 countries found that Nepal ranked 22nd out of the 30 countries on ECD performance. Furthermore, Nepal was among the worst performing countries regarding ECD indicators in South Asia, underscoring the need to prioritise ECD nationally (United Nations Children's Fund Nepal, 2018). To our knowledge, no study has examined the association of malnutrition and ECD outcomes in Nepal.

In this study, we focus on the role of nutrition as one component within the Nurturing Care Framework and assess the effects of stunting, wasting, and underweight on ECD in the domains of literacy and numeracy, physical development, socio‐emotional development, and learning skills (and examine malnutrition's effects on ECD index parameters overall) among children 36–59 months of age using the 2019 Nepal Multiple Indicator Cluster Survey (NMICS) data set (Government of Nepal National Planning Commission Central Bureau of Statistics & UNICEF Nepal, 2020).

## 2 METHODS

This study uses the sixth (most recent) round of the Nepal Multiple Indicator Cluster Survey (NMICS) data set, a nationally representative cross‐sectional household survey conducted by the Central Bureau of Statistics in collaboration with the United Nations Children's Fund (UNICEF), as part of the Global MICS Programme (UNICEF, 2021). The data set includes a wide range of household socio‐demographic characteristics as well as children's health and nutritional status. Sample households were selected in two stages. During the first stage, the number of census enumeration areas (clusters) were selected systematically using probability proportional to size sampling. In the second stage, clusters were selected using a systematic random sampling method, where 25 households with or without children (below the age of 5 as well as at or above the age of 5) were selected from each cluster. The survey's sampling processes are documented in the full MICS report (Government of Nepal National Planning Commission Central Bureau of Statistics & UNICEF Nepal, 2020).

In the 2019 NMICS survey, of the total 12,655 households interviewed, height and weight information was collected from 6656 children. Of these children, 2871 were 36–59 months old. Although valid tools that assess childhood development before 36 months of age exist, the latest ECD tools used in the MICS survey are restricted to children 36–59 months of age due to time and resource constraints as well as limited comparable measurement tools for children under 36 months of age (Loizillon et al., 2017).

Stunting, wasting and underweight indicators were used to measure childhood nutritional status. Based on the WHO growth standard, stunting was measured based on height‐for‐age z‐scores (≤2 standard deviation). Wasting was measured based on child weight‐for‐height z‐scores (≤2 standard deviation) and underweight was measured based on child weight‐for‐age z‐scores (≤2 standard deviation) (Crowe et al., 2014).

According to the World Health Organization's (WHO) guidelines, outliers for child anthropometry were excluded, including length/height‐for‐age z‐scores (>6 or ≤6) (*n *= 44), weight‐for‐age z‐scores (>5 or ≤5) (*n* = 40), and weight‐for‐length/height z‐scores (>5 or ≤6) (*n* = 11) (Crowe et al., 2014). The final sample size for this analysis consisted of 2826 children who were identified as stunted, 2831 children as wasted and 2859 children as underweight.

The ECD outcome variable (from the ECD index) was measured based on composites of four domains including literacy‐numeracy, physical, social‐emotional and learning development. A 10‐item module was used in the MICS survey to calculate the ECD index. The index is designed for children 36–59 months of age (the age of children in our study sample) to assess the four domains of ECD (L. Richter et al., 2019) and is based on selected milestones that children are expected to achieve by 36–59 months. A child is considered developmentally on track for the literacy‐numeracy domain if they exhibit at least two of the following three learning behaviours: identifying and naming at least 10 letters of the alphabet, reading at least four simple common words, and knowing the names and symbols of numbers from 1 to 10. Similarly, a child is considered on track for physical development if they can pick up a small object with two fingers, such as a stick or a rock, or if the child's mother or caretaker indicate that the child is able to play. A child is aligned with adequate social‐emotional development if they exhibit at least two of the following behaviours: getting along well with other children, refraining from kicking, biting, or hitting other children, and not getting distracted easily. Likewise, children's approaches to learning are considered on track if children exhibit either one or both behaviours: ability to follow simple directions on how to perform a task and performing a task independently. Finally, the NMICS survey's ECD index was calculated based on percentages of children who were considered developmentally on track in at least three of the four domains (literacy‐numeracy, physical, social‐emotional and learning development) (Loizillon et al., 2017).

We conducted a disaggregated analysis of household characteristics and child's nutrition status to explore associations of child nutritional status with the four domains of the ECD index (literacy‐numeracy, physical, social‐emotional and learning development). Using an adjusted logistic regression analysis, researchers adjusted socio‐demographic confounders such as wealth quintile, caste and ethnicity, head of households, family size, age and gender of the child and mother's education level based on national context and prior studies (Adhikari et al., 2019; Sunuwar et al., 2020). For wealth, we used the NMICS survey's quintiles, based on ownership of durable goods, household characteristics and basic services (Martel, 2016; Rutstein & Staveteig, 2014). The caste and ethnicity variable were classified into four different groups: Brahmin/Chhetri, Janajaties, Dalit, and other. Heads of households were categorised as male‐headed or female‐headed. Family size was aggregated into two groups: less than five and five or more members. Finally, mother's education level was classified into four categories: no education, below secondary, secondary and above secondary level. Confounding variables such as access to and enrolment in ECD programmes, access to books and playing resources, maternal nutrition, breastfeeding, and complementary feeding, were not assessed in this study (Huang et al., 2016; Makrides et al., 2011; Pérez‐Escamilla & Hall Moran, 2016; United Nations Children's Fund Nepal, 2018; Veena et al., 2016). Analyses were performed by applying sampling weights for national representation and were conducted in STATA version 14 (Stata Statistical Software: Release 14, 2015).

## 3 RESULTS

### 3.1 General demographics

The study included a total of 2871 children ages 36–59 months (see Table 1 for the general demographics). One‐third of mothers completed basic and secondary education and over a quarter of mothers had no formal education. The families of children ages 36–59 months were almost equally distributed across wealth quintiles (lowest quintile, second, third, and fourth), with one‐fifth of households in each one. Sixty‐five percent of families of children 36–59 months had five or more family members in their household. Three‐fourths of households were led by a male. Nearly three quarters (73.8%) of households belonged to the two upper Janajati (42.1%) and Bhramin/Chhetri (31.7%) castes.

**Table 1:** General demographics

Demographics	%	No.
Mother's education		
None	28.5	817
Basic (Gr 1–8)	33.4	959
Secondary (Gr 9–12)	31.5	905
Higher	6.6	190
Total	100.0	2871
Wealth index quintile		
First (lowest)	22.7	652
Second	20.3	584
Middle	20.6	590
Fourth	20.0	573
Fifth (highest)	16.4	472
Total	100.0	2871
Number of members per household		
Less than five	34.7	996
Five and above	65.3	1875
Total	100.0	2871
Caste of household		
Brahmin/Chhetri	31.7	909
Janajaties	42.1	1209
Dalit	18.0	517
Other	8.2	236
Total	100.0	2871
Sex of household head		
Male	73.3	2105
Female	26.7	766
Total	100.0	2871

Among children 36–59 months of age, 35.7% had low height for their age, 25.8% were underweight, and 9.3% suffered from acute malnutrition. Children's ECD status were mostly on track for physical development (96.7%) and learning development (90.4%). However, 35% were not considered developmentally on track for the ECD index (a composite indicator for early childhood development [ECD]).

### 3.2 Early childhood development indicators

Children with or without wasting did not demonstrate a statistically significant difference in ECD indicators; however, the proportion of children considered developmentally on‐track was inversely associated with those who exhibited stunting or underweight. Stunting and underweight were also inversely associated with the four domains of the ECD index (literacy‐numeracy, physical, social‐emotional and learning development). The proportion of children who were developmentally on‐track was higher when household wealth status increased. In the caste and ethnicity category, Dalit children were less likely to be developmentally on track compared to those from Bhramin/Chhetri and Janajati castes. Mother's education levels were positively associated with children's development status, with higher education levels corresponding to children being more developmentally on‐track.

### 3.3 Association of early childhood development status with child nutritional status

**Figure 1:**
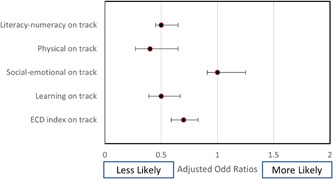
Forest plot depicting association between stunting and early childhood development

**Figure 2:**
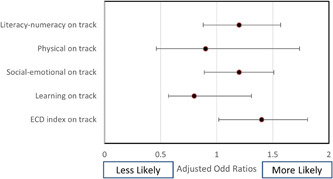
Forest plot depicting association between wasting and early childhood development

**Figure 3:**
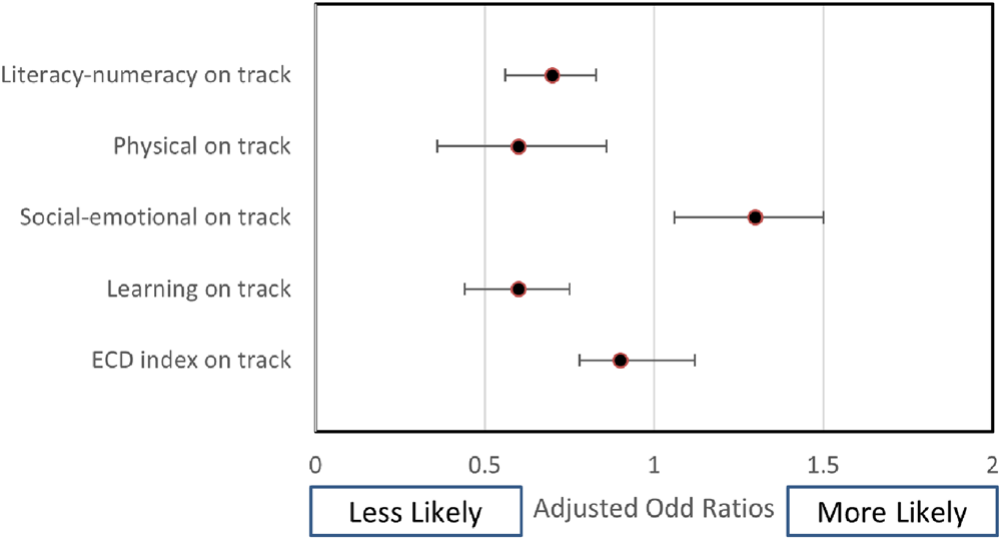
Forest plot depicting association between underweight and early childhood development

To identify associations between ECD indicators and prominent forms of malnutrition, data in the logistic regression model was adjusted for individual and household characteristics, including household wealth quintile, family size, sex of the head of household, caste and ethnicity, mother's level of education, and gender and age of the child. Forest plots in Figures 1, 2, 3 present the association of different forms of malnutrition with ECD indicators.

Adjusted odds ratios indicated that stunted children were 0.68 times less likely (*p* < 0.01, CI: 0.57–0.8) to be developmentally on track for the ECD index and for the three domains of ECD: literacy‐numeracy (OR 0.49, *p* < 0.01, CI: 0.41–0.60), physical development (OR 0.4, *p* < 0.01, CI: 0.27–0.64) and learning development (OR 0.51, *p* < 0.01, CI: 0.39–0.66). Similarly, children who were underweight had lower odds of being on track for literacy‐numeracy (0.64, *p* < 0.01, CI: 0.52–0.79), physical (OR 0.56, *p* < 0.01, CI: 0.36–0.85) and learning (OR 0.56, *p* < 0.01, CI: 0.43–0.74) domains of the ECD index.

Neither stunting nor underweight was associated with socioemotional development of the early childhood domain. Moreover, wasting did not demonstrate statistically significant associations with any of the child development indicators. Notably, children who were both stunted and wasted were 0.80 times less likely to be developmentally on track for ECD index had lower odds of being on track for literacy numeracy (OR 0.63, *p* < 0.01, CI: 0.52–0.75), physical development (OR 0.4, *p* < 0.01, CI: 0.31–0.75) and learning development (OR 0.62, *p* < 0.01, CI: 0.48–0.80).

Confounding variables such as household wealth quintile, mother's level of education, and child's age in months had statistically significant associations with child development indicators. For instance, high household wealth quintiles were associated with increased odds of being developmentally on track with the ECD index (OR 2.3–2.5). Highest associations occurred between wealth quintile and literacy‐numeracy (OR 5.2–6.0 for the fifth wealth quintile). Likewise, a mother's high level of education correlated with increased odds of being developmentally on track with the ECD index (OR 3.4–3.7 for above secondary level of education), with highest associations observed between literacy‐numeracy and mothers with above secondary level of education (OR 6.6–7.1). Compared to male‐headed households, children in families with female household heads were more likely to be developmentally on track for two of the four domains of the ECD index and overall ECD index (OR 1.30, *p* < 0.01, CI: 1.06–1.59) for stunted and (OR 1.29, *p* < 0.01, CI: 1.06–1.58) for wasted children. Children with large family size (five and above) were less likely to be developmentally on track for at least two of the parameters of ECD index among stunted and underweight children. There were no significant differences in ECD parameters by child gender among stunted, wasted and underweight children. However, findings suggest that female children aged 36–59 months were more likely to be on track regarding socio‐emotional development among children who were stunted (OR 1.19, *p* < 0.05, CI: 1.02–1.39), wasted (OR 1.20, *p* < 0.05, CI: 1.04–1.40) and underweight (OR 1.20, *p* < 0.05, CI: 1.03–1.39). Our study also demonstrated that ECD outcomes improve as child's age increased in months. The domains of literacy numeracy (OR 1.11, *p* < 0.01, CI: 1.10–1.13), physical development (OR 1.04, *p* < 0.01, CI: 1.01–1.07) and learning development (OR 1.03, *p* < 0.01, CI: 1.01–1.05) improved as child's age increased in months among stunted children.

Associations between ECD domains and malnutrition indicators of stunting, wasting and underweight are presented in the **Supplementary** Appendix.

## 4 DISCUSSION

Our study found that 35.7%, 25.8% and 9.3% of children 36–59 months were stunted, underweight and wasted, respectively (*N* = 2871) and that approximately 35% of Nepali children (1004 children) were developmentally off track for the ECD index. These results align with other studies in South Asia, where 27.8 million children 36–59 months failed to attain ECD benchmarks (McCoy et al., 2016). However, previous literature has demonstrated that not all items on the ECD Index are age‐appropriate, and therefore may overestimate children's developmental competence (McCoy et al., 2016).

Malnutrition and its various manifestations are an urgent and enduring public health issue in Nepal (Bhattarai et al., 2017; Budhathoki et al., 2020), resulting in delays in various ECD domains and potentially leading to permanent developmental impairments (Groce et al., 2014; Pravana et al., 2017). We used a population‐based data set to analyse childhood nutrition status and its association with early childhood indicators. Poor childhood development outcomes were concentrated among children who were stunted, underweight, wasted, poor and who had mothers with low educational status, and lived in male‐headed households. Our study demonstrates that children who were stunted or underweight had lower odds of achieving the ECD index for literacy‐numeracy, physical and learning domains.

Studies have shown that environmental stimulation, particularly responsive and stimulating caregiving, high quality of care, and child interaction, can promote children's early cognitive development despite the presence of risk factors such as poverty and malnutrition (Nores & Barnett, 2010; Walker et al., 2005). Combining child development activities such as responsive caregiving, psychological and educational interventions, and high quality care along with services for undernourished children would benefit child growth and development (S. M. Grantham‐McGregor et al., 2014). For instance, a population level analysis in South Asian countries (Bangladesh, Bhutan, Nepal and Pakistan) revealed that children who were stunted and underweight had poor ECD outcomes (Kang et al., 2018). A recent multi‐country cohort study in Nepal, Bangladesh, India, Brazil and Peru demonstrated the association between early‐onset persistent stunting and lower cognitive development among children 5 years of age (Alam et al., 2020). Furthermore, a study conducted in Tanzania revealed that height for age had a positive linear association with cognitive, communicative and motor development skills (Sudfeld et al., 2015). Evidence generated through meta‐analyses in low‐ and middle‐income countries (LMICs) supports the association between stunting and poor childhood development, including developmental markers such as adequate motor skills, socio‐emotional competencies and learning milestones (Perkins et al., 2017). Furthermore, each unit increase in height‐for‐age z‐scores for children ≤2 years old was associated with an increase in cognitive abilities from 5 to 11 years in a meta‐analysis in LMICs (Sudfeld et al., 2015). At least 249 million (43%) children under the age of 5 in LMICs are at risk of poor ECD outcomes as a consequence of being stunted or living in extreme poverty, with the highest proportions in South Asia and sub‐Saharan Africa (Black et al., 2017).

Notably, our study found no significant association between wasting and ECD indicators. This aligns with a similar study conducted in Bangladesh, Bhutan, Nepal, Pakistan, Afghanistan and two provinces of India, where wasting had no significant relationship with childhood development in all countries except Pakistan (Kang et al., 2018). A study conducted in Bangladesh found wasted children had poor motor skills (gross motor and total motor development) compared to nonwasted peers (Nahar et al., 2020). Very limited studies were found to reveal an association between childhood wasting and ECD parameters, providing an opportunity for further investigation. Similarly, no association was observed with socio‐emotional development and malnutrition indicators in our study. Our findings align with another study conducted in South Asia, where stunting (OR 0.99, 95% CI: 0.92–1.07), underweight (OR 1.05, 95% CI: 0.97–1.14) and wasting (OR 1.07, 95% CI: 0.86–1.33) were not associated with the social–emotional ECD domain in the pooled sample (Kang et al., 2018).

According to our study, stunting, which begins in utero and is exacerbated during the first 1000 days of life, and low weight for age (underweight) that may stem from recent, acute weight loss, have adverse ECD implications, especially for learning, physical and numeracy domains. These deleterious associations could impede future academic performance and economic success (S. Grantham‐McGregor et al., 2007). According to recent World Bank estimates, the average country's gross domestic product per capita would be 7% higher had stunting been eliminated when today's adults were children (Galasso & Wagstaff, 2019). Health and nutrition interventions such as good antenatal care, breastfeeding and complementary feeding programmes and counselling, growth monitoring, immunisation and antenatal micronutrient supplementation have shown to be effective in attenuating stunting and underweight and could have a positive impact on early childhood in Nepal and elsewhere (Hossain et al., 2017; Keats et al., 2021). To achieve sufficient development in a child's early years, adequate nutrition and care, responsiveness and stimulation are crucial (Hanley‐Cook et al., 2020). However, nutrition programmes are often focused on the first 1000 days while ECD interventions prioritise children 3–6 years of age.

The Nurturing Care Framework recognises the importance of appropriate health care, nutrition, safety and security, and responsive caregiving during a child's early years for optimal child development. As per this framework, integrating ECD interventions into nutrition programming during the first 1000 days of life through the child's fifth birthday would contribute toward achieving the United Nations' Sustainable Development Goals for child health (UNICEF & World Health Organization, 2012; United Nations Department of Economic and Social Affairs Sustainable Development, 2021). In Nepal, ECD indicator performance ranks among the worst in South Asia. Our findings suggest that ECD indicators are poor among children who are stunted, underweight, and both stunted and wasted (though there is no evidence that stunting delays ECD), potentially due to the common determinants that ECD and poor child development outcomes both share, such as poor nutrition, inadequate care and repeated infections (Leroy & Frongillo, 2019). Therefore, interventions that address the common determinants of malnutrition and ECD in early years could improve ECD outcomes among young children later. Given similar contexts in the region, addressing the common determinants to promote overall childhood development and responsive caregiving among malnourished children could help South Asian countries meet the United Nations Sustainable Development Goals 3 and 4.2.

Our findings highlight the socio‐demographic factors associated with ECD outcomes and malnutrition and highlight the importance of targeting interventions to children most adversely impacted. When we adjusted for confounding variables, household wealth quintile had a positive linear association across various domains of ECD indicators in addition to nutritional status. The proportion of children who were on track with ECD indicators increased with higher household wealth quintiles, which aligns with previous, similar studies. In one study conducted in LMICs between 2010 and 2018, on average, household wealth disparities were observed to be a predictor of ECD outcomes in almost all regions and countries (Lu et al., 2020). Children in the lowest quintile were consistently scoring worse on all four ECD indicators compared to peers in the highest quintile. An analysis conducted in four developing countries on mediators that potentially affected the association between child cognitive development and their households' socioeconomic status demonstrated that early nutrition, urban residence, and caregiver's education served as significant mediators, though the magnitude of their effects varied (Lopez Boo, 2016).

A regional study of eight countries in West and Central Africa demonstrated that disparities in ECD indicators were observed based on children's household wealth quintiles (29% difference between the lowest and highest wealth quintiles) and mother's level of education (20% difference between ‘less than secondary' and ‘secondary and higher' levels of education). Using a linear regression model, household wealth was among the strongest predictors of whether children were developmentally on track (Loizillon et al., 2017). A study conducted in 15 countries with available 2010–2011 MICS data demonstrated that low maternal education levels were associated with increased risk of poor ECD outcomes among children (L. M. Richter et al., 2017). Globally, human capital accounts for almost two‐thirds of wealth differences between countries (Lange et al., 2018) and ECD is the foundation of human capital. Interventions targeting low‐income households focusing on socioeconomic barriers to child development are necessary to mitigate economic disparities and ensure that quality, accessible and affordable care is available to the most vulnerable children.

Our study suggests that ECD outcomes improve as a child's age increases in months irrespective of stunting, wasting or underweight. Limited studies exist that demonstrate the potential association of ECD parameters with child's age among children who are stunted, wasted and underweight. More research is necessary to observe the association of ECD outcomes and malnutrition and design age‐appropriate interventions to improve child development outcomes. However, our study found no disparity in ECD outcomes among male and female children, unlike other studies in low‐ and middle‐income countries in 2005–2015 (McCoy et al., 2016) which observed positive associations between low development scores and male sex. Nevertheless, DHS analysis of 135 countries from 2010 to 2018 found negligible gaps between boys and girls with ECD outcomes (Lu et al., 2020).

## 5 STRENGTHS AND LIMITATIONS

The ECD period spans from birth through the first 8 years of life and is critical to a child's cognitive, social, emotional and physical development (Inequities in ECD What the data say: Evidence from the Multiple Indicator Cluster Surveys, 2012). However, limited by the NMICS 2019 data set, ECD indicators could only be assessed for children aged 36–59 months and thus underestimate the true burden of developmental challenges in low‐resource settings such as Nepal. The ECD index is a composite index within the MICS and thus cannot be used more broadly for SDG monitoring purposes (United Nations Sustainable Development Solutions Network SDSN, 2021). Other ECD indicators, such as access to and enrolment in ECD programmes, access to books and playing resources, as well as nutrition indicators, including dietary diversity, breastfeeding, and mother's nutrition status were beyond the scope of this study and could be explored in future studies. The lack of association between wasting and ECD indicators could be due to the low prevalence of wasting within the study's sample size and also because wasting incidents are transitory (compared to the cumulative effects of stunting); future studies could explore wasting and its implications on ECD outcomes and investigate beyond statistical significance to determine associations (Ciapponi et al., 2021). Despite these limitations, this study's findings are comparable to findings in similar settings. Further, since the MICS survey methodology is standardised globally, our study method can be replicated in other countries.

## 6 CONCLUSION

Improving ECD is crucial for meeting the United Nations' Sustainable Development Goals (Pérez‐Escamilla & Moran, 2017). This study's findings demonstrate that stunting and underweight could serve as strong predictors of ECD progress among children 36–59 months of age. While nutrition‐specific interventions are essential for child development, they alone are insufficient in supporting children to reach full developmental potential (Black et al., 2017). Targeted ECD interventions within nutrition programmes among children who are stunted, underweight, have mothers with limited education, and are from poor households are necessary to foster overall childhood development.

## CONFLICT OF INTERESTS

The authors declare that there are no conflict of interests.

## AUTHOR CONTRIBUTIONS

Manisha L. Shrestha performed the research, designed the research study, and wrote the first draught of the study. Kelly E. Perry provided major revisions and served as the paper's key technical reviewer. Ramesh P. Adhikari assisted in analysing the data and Basant Thapa, Ramesh P. Adhikari and Amy Weissman provided study revisions and reviewed content for technical accuracy.

## Supporting information

Supporting information.

## Data Availability

The data that support the findings of this study are openly available in MICS data set at https://mics.unicef.org/surveys
