# Supplementary material for: Malnutrition matters: Association of stunting and underweight with early childhood development indicators in Nepal
Source: Matern Child Nutr. 2022 Jan 20;18(2):e13321. doi: 10.1111/mcn.13321 (PMC8932687; doi:10.1111/mcn.13321)
Supplement: Supplementary file 1 — Supporting information. [file MCN-18-e13321-s001.docx]

**Supplementary Appendix**

**Table 2: Early Childhood Development Status by Demographics**

|  | **Literacy-numeracy on track** | **Physical on track** | **Social-emotional development on track** | **Learning development on track** | **ECD index on track** | **No.** |
| --- | --- | --- | --- | --- | --- | --- |
| **Child nutritional status** | | | | | | |
| Stunting† | | | | | | |
| No | 48.2 | 97.9 | 55.9 | 93 | 69.2 | 1818 |
| Yes | 27.6 | 94.5 | 55.9 | 86.4 | 56.6 | 1009 |
| *Total* | | | | | | 2827 |
| Wasting† | | | | | | |
| No | 40.7 | 96.8 | 55.6 | 90.8 | 64.2 | 2569 |
| Yes | 40 | 96 | 58.9 | 89.3 | 68.6 | 262 |
| *Total* | | | | | | 2831 |
| Underweight† | | | | | | |
| No | 44.8 | 97.4 | 55 | 91.9 | 66.4 | 2123 |
| Yes | 28.4 | 94.6 | 58.7 | 86.3 | 59.3 | 737 |
| *Total* | | | | | | 2860 |
| **Wealth quintile** | | | | | | |
| First (lowest) | 19.7 | 95.5 | 53.5 | 86 | 52 | 652 |
| Second | 30.2 | 97 | 56.9 | 91 | 60.2 | 584 |
| Third | 39.3 | 97.4 | 57.1 | 92.4 | 66.7 | 590 |
| Fourth | 48.8 | 95.1 | 51.7 | 91.4 | 66.7 | 573 |
| Fifth (highest) | 73 | 98.9 | 62.1 | 91.7 | 81.6 | 472 |
| **Caste/ethnicity** | | | | | | |
| Brahmin/chhetri | 45.7 | 97.2 | 56 | 91.3 | 68.5 | 909 |
| Janajaties | 42.9 | 97.8 | 56.9 | 89.8 | 65.6 | 1209 |
| Dalit | 31.6 | 95.3 | 58.2 | 89.9 | 62.4 | 517 |
| other | 26.9 | 92 | 46.4 | 90.8 | 47.9 | 236 |
| **Sex of head of household** | | | | | | |
| Male | 36.8 | 96.6 | 54.4 | 91 | 62 | 2105 |
| Female | 50.5 | 97 | 60.4 | 88.6 | 71.5 | 766 |
| **Family size** | | | | | | |
| less than 5 | 50.7 | 97.5 | 60.2 | 90.6 | 71.9 | 996 |
| 5 and above | 35 | 96.2 | 53.7 | 90.2 | 60.6 | 1875 |
| **Mother’s education** | | | | | | |
| None | 18.5 | 96.1 | 52.7 | 87.9 | 50.5 | 817 |
| Basic (grade 1-8) | 37.5 | 96.5 | 55.1 | 91.5 | 63.7 | 959 |
| Secondary (grade 9-12) | 55.8 | 96.8 | 60 | 90.7 | 73.7 | 905 |
| Above secondary | 76.4 | 99.2 | 55.5 | 93.3 | 85.1 | 190 |
| **Gender of the child** | | | | | | |
| Boy | 41.9 | 96.5 | 54.1 | 90.5 | 65.1 | 1565 |
| Girl | 38.7 | 96.9 | 58.2 | 90.2 | 63.8 | 1306 |
| **Age of the child** | | | | | | |
| 3 years | 27.6 | 96.1 | 54.3 | 88.2 | 56.3 | 1468 |
| 4 years | 53.8 | 97.3 | 57.7 | 92.6 | 73.1 | 1403 |
| *Total* | 40.4 | 96.7 | 56 | 90.4 | 64.5 | 2871 |

† Outliers were excluded so the category’s sample size is less than the study’s total sample size (N=2871).

**Table 3. Association between Early Childhood Development Status and Stunting**

|  | **Literacy-numeracy on track** | **Physical on track** | **Social-emotional on track** | **Learning on track** | **ECD index on track** |
| --- | --- | --- | --- | --- | --- |
|  | **OR/P (CI)** | **OR/P (CI)** | **OR/P (CI)** | **OR/P (CI)** | **OR/P (CI)** |
| Stunted | 0.49** [0.41 - 0.60] | 0.41** [0.27 - 0.64] | 1.06 [0.90 - 1.24] | 0.51** [0.39 - 0.66] | 0.68** [0.57 - 0.81] |
| **Wealth quintile** | | | | | |
| First (lowest) (R) |  |  |  |  |  |
| Second | 1.61** [1.21 - 2.15] | 1.52 [0.81 - 2.85] | 1.17 [0.93 - 1.48] | 1.54* [1.06 - 2.25] | 1.34* [1.05 - 1.70] |
| Third | 2.40** [1.80 - 3.19] | 1.64 [0.86 - 3.13] | 1.13 [0.90 - 1.43] | 1.77** [1.20 - 2.63] | 1.71** [1.34 - 2.19] |
| Fourth | 3.00** [2.24 - 4.01] | 1.00 [0.55 - 1.83] | 0.88 [0.69 - 1.13] | 1.51* [1.01 - 2.27] | 1.48** [1.14 - 1.92] |
| Fifth (highest) | 5.25** [3.77 - 7.32] | 2.58 [0.94 - 7.13] | 1.33* [1.01 - 1.76] | 1.32 [0.82 - 2.12] | 2.29** [1.65 - 3.17] |
| **Caste/ethnicity** | | | | | |
| Brahmin/Chhetri (R) |  |  |  |  |  |
| Janajaties | 0.90 [0.73 - 1.11] | 1.23 [0.69 - 2.18] | 1.01 [0.85 - 1.21] | 0.79 [0.58 - 1.08] | 0.89 [0.73 - 1.09] |
| Dalit | 0.78 [0.60 - 1.03] | 0.63 [0.35 - 1.14] | 1.19 [0.95 - 1.50] | 0.92 [0.62 - 1.35] | 1.03 [0.81 - 1.32] |
| Other | 0.48** [0.33 - 0.70] | 0.31** [0.16 - 0.62] | 0.73* [0.54 - 1.00] | 0.84 [0.49 - 1.44] | 0.48** [0.35 - 0.67] |
| **Sex of head of household** | | | | | |
| Male (R) |  |  |  |  |  |
| Female | 1.57** [1.27 - 1.94] | 0.97 [0.58 - 1.61] | 1.17 [0.98 - 1.40] | 0.68** [0.51 - 0.91] | 1.30** [1.06 - 1.59] |
| **Household size** | | | | | |
| Less than five (R) |  |  |  |  |  |
| Five and above | 0.78* [0.64 - 0.95] | 0.74 [0.45 - 1.24] | 0.84 [0.71 - 0.99] | 0.93 [0.70 - 1.25] | 0.77** [0.64 - 0.93] |
| **Mother’s education level** | | | | | |
| None (R) |  |  |  |  |  |
| Basic (grade 1-8) | 2.11** [1.65 - 2.70] | 0.94 [0.56 - 1.59] | 1.06 [0.87 - 1.30] | 1.35 [0.97 - 1.88] | 1.48** [1.20 - 1.82] |
| Secondary (grade 9-12) | 3.75** [2.89 - 4.86] | 0.78 [0.43 - 1.39] | 1.25* [1.01 - 1.55] | 1.13 [0.79 - 1.61] | 2.08** [1.65 - 2.62] |
| Above secondary | 6.61** [4.22 - 10.36] | 3.99 [0.42 - 37.58] | 0.99 [0.69 - 1.43] | 1.52 [0.75 - 3.07] | 3.46** [2.15 - 5.58] |
| **Child Sex** | | | | | |
| Boy ® |  |  |  |  |  |
| Girl | 0.94 [0.78 - 1.12] | 1.18 [0.77 - 1.80] | 1.19* [1.02 - 1.39] | 0.96 [0.74 - 1.24] | 0.98 [0.83 - 1.15] |
| **Child age in months** | 1.11** [1.10 - 1.13] | 1.04** [1.01 - 1.07] | 1.01* [1.00 - 1.02] | 1.03** [1.01 - 1.05] | 1.07** [1.05 - 1.08] |

* p < 0.05; ** p < 0.01

**Table 4. Association between Early Childhood Development Status and Wasting**

|  | **Literacy-numeracy on track** | **Physical on track** | **Social-emotional on track** | **Learning on track** | **ECD index on track** |
| --- | --- | --- | --- | --- | --- |
|  | **OR/P (CI)** | **OR/P (CI)** | **OR/P (CI)** | **OR/P (CI)** | **OR/P (CI)** |
| Wasted | 1.27 [0.94 - 1.72] | 0.92 [0.47 - 1.79] | 1.18 [0.91 - 1.54] | 0.87 [0.57 - 1.33] | 1.41* [1.05 - 1.88] |
| **Wealth quintile** | | | | | |
| First (lowest) (R) |  |  |  |  |  |
| Second | 1.72** [1.29 - 2.29] | 1.72 [0.92 - 3.21] | 1.15 [0.91 - 1.45] | 1.62 [1.12 - 2.36] | 1.38** [1.09 - 1.76] |
| Third | 2.50** [1.89 - 3.31] | 1.87 [0.99 - 3.56] | 1.13 [0.90 - 1.42] | 1.87** [1.27 - 2.76] | 1.75** [1.37 - 2.23] |
| Fourth | 3.11** [2.33 - 4.15] | 1.15 [0.63 - 2.09] | 0.90 [0.70 - 1.15] | 1.62* [1.08 - 2.43] | 1.53** [1.18 - 1.99] |
| Fifth (highest) | 6.02** [4.33 - 8.36] | 3.38* [1.23 - 9.24] | 1.35* [1.02 - 1.78] | 1.55 [0.97 - 2.48] | 2.50** [1.81 - 3.45] |
| **Caste/ethnicity** | | | | | |
| Brahmin/Chhetri (R) |  |  |  |  |  |
| Janajaties | 0.92 [0.74 - 1.13] | 1.23 [0.69 - 2.17] | 1.02 [0.85 - 1.22] | 0.84 [0.62 - 1.14] | 0.91 [0.74 - 1.11] |
| Dalit | 0.78 [0.59 - 1.02] | 0.62 [0.34 - 1.12] | 1.18 [0.94 - 1.48] | 0.93 [0.63 - 1.36] | 0.99 [0.77 - 1.27] |
| Other | 0.49** [0.34 - 0.71] | 0.31** [0.15 - 0.60] | 0.74 [0.55 - 1.00] | 0.87 [0.51 - 1.48] | 0.49** [0.36 - 0.68] |
| **Sex of head of household** | | | | | |
| Male (R) |  |  |  |  |  |
| Female | 1.54** [1.25 - 1.90] | 0.93 [0.56 - 1.55] | 1.17 [0.98 - 1.40] | 0.68** [0.51 - 0.90] | 1.29* [1.05 - 1.57] |
| **Household size** | | | | | |
| Less than five (R) |  |  |  |  |  |
| Five and above | 0.77** [0.63 - 0.93] | 0.72 [0.43 - 1.21] | 0.85 [0.72 - 1.01] | 0.96 [0.72 - 1.28] | 0.78** [0.64 - 0.94] |
| **Mother’s education level** | | | | | |
| None (R) |  |  |  |  |  |
| Basic (grade 1-8) | 2.20** [1.72 - 2.81] | 0.95 [0.56 - 1.60] | 1.08 [0.89 - 1.31] | 1.40* [1.01 - 1.94] | 1.54** [1.26 - 1.90] |
| Secondary (grade 9-12) | 4.02** [3.10 - 5.20] | 0.83 [0.46 - 1.48] | 1.25* [1.01 - 1.55] | 1.25 [0.88 - 1.78] | 2.19** [1.74 - 2.76] |
| Above secondary | 7.14** [4.57 - 11.17] | 4.19 [0.45 - 39.40] | 0.98 [0.68 - 1.41] | 1.66 [0.83 - 3.35] | 3.70** [2.30 - 5.95] |
| **Child Sex** | | | | | |
| Boy ® |  |  |  |  |  |
| Girl | 0.94 [0.79 - 1.12] | 1.18 [0.77 - 1.80] | 1.20* [1.04 - 1.40] | 0.95 [0.74 - 1.23] | 0.98 [0.83 - 1.16] |
| **Child age in months** | 1.11** [1.10 - 1.13] | 1.04* [1.01 - 1.07] | 1.01* [1.00 - 1.02] | 1.03** [1.01 - 1.05] | 1.07** [1.05 - 1.08] |

* p < 0.05; ** p < 0.01

**Table 5. Association between Early Childhood Development Status and Underweight**

|  | **Literacy-numeracy on track** | **Physical on track** | **Social-emotional on track** | **Learning on track** | **ECD index on track** |
| --- | --- | --- | --- | --- | --- |
|  | **OR/P (CI)** | **OR/P (CI)** | **OR/P (CI)** | **OR/P (CI)** | **OR/P (CI)** |
| Underweight | 0.64** [0.52 - 0.79] | 0.56** [0.36 - 0.85] | 1.26** [1.05 - 1.50] | 0.56** [0.43 - 0.74] | 0.91 [0.75 - 1.09] |
| **Wealth quintile** | | | | | |
| First (lowest) (R) |  |  |  |  |  |
| Second | 1.71** [1.29 - 2.28] | 1.62 [0.87 - 3.03] | 1.16 [0.92 - 1.46] | 1.61** [1.11 - 2.33] | 1.39** [1.10 - 1.77] |
| Third | 2.54** [1.91 - 3.36] | 1.82 [0.95 - 3.45] | 1.14 [0.91 - 1.43] | 1.90** [1.29 - 2.79] | 1.78** [1.40 - 2.28] |
| Fourth | 2.97** [2.23 - 3.97] | 1.05 [0.58 - 1.90] | 0.91 [0.71 - 1.16] | 1.51* [1.01 - 2.25] | 1.53** [1.18 - 1.98] |
| Fifth (highest) | 5.53** [3.98 - 7.69] | 2.98* [1.08 - 8.20] | 1.39* [1.05 - 1.83] | 1.28 [0.80 - 2.03] | 2.38** [1.73 - 3.28] |
| **Caste/ethnicity** | | | | | |
| Brahmin/Chhetri (R) |  |  |  |  |  |
| Janajaties | 0.90 [0.73 - 1.11] | 1.14 [0.65 - 2.02] | 1.03 [0.86 - 1.23] | 0.75 [0.55 - 1.02] | 0.89 [0.73 - 1.08] |
| Dalit | 0.79 [0.61 - 1.04] | 0.63 [0.35 - 1.13] | 1.17 [0.93 - 1.47] | 0.93 [0.63 - 1.36] | 1.00 [0.78 - 1.28] |
| Other | 0.51** [0.35 - 0.74] | 0.32** [0.16 - 0.64] | 0.73* [0.54 - 0.98] | 0.87 [0.51 - 1.47] | 0.48** [0.35 - 0.67] |
| **Sex of head of household** | | | | | |
| Male (R) |  |  |  |  |  |
| Female | 1.52** [1.23 - 1.87] | 0.92 [0.55 - 1.54] | 1.18 [0.99 - 1.42] | 0.68** [0.51 - 0.90] | 1.29** [1.06 - 1.58] |
| **Household size** | | | | | |
| Less than five (R) |  |  |  |  |  |
| Five and above | 0.78* [0.64 - 0.95] | 0.73 [0.43 - 1.21] | 0.84* [0.71 - 1.00] | 0.93 [0.70 - 1.24] | 0.77** [0.64 - 0.93] |
| **Mother’s education level** | | | | | |
| None (R) |  |  |  |  |  |
| Basic (grade 1-8) | 2.16** [1.69 - 2.75] | 0.89 [0.53 - 1.49] | 1.07 [0.88 - 1.30] | 1.41* [1.02 - 1.95] | 1.52** [1.24 - 1.87] |
| Secondary (grade 9-12) | 3.91** [3.03 - 5.06] | 0.80 [0.45 - 1.44] | 1.26* [1.02 - 1.57] | 1.24 [0.87 - 1.76] | 2.17** [1.73 - 2.73] |
| Above secondary | 6.98** [4.46 - 10.90] | 3.91 [0.42 - 36.72] | 0.99 [0.69 - 1.43] | 1.72 [0.86 - 3.47] | 3.68** [2.29 - 5.91] |
| **Child Sex** | | | | | |
| Boy ® |  |  |  |  |  |
| Girl | 0.93 [0.78 - 1.11] | 1.20 [0.79 - 1.83] | 1.20* [1.03 - 1.39] | 0.98 [0.76 - 1.27] | 0.98 [0.83 - 1.16] |
| **Child age in months** | 1.11** [1.10 - 1.13] | 1.04* [1.00 - 1.07] | 1.01* [1.00 - 1.02] | 1.03** [1.02 - 1.05] | 1.07** [1.05 - 1.08] |

* p < 0.05; ** p < 0.01

**Table 6. Association between Early Childhood Development Status and Stunting+Wasting**

|  | **Literacy-numeracy on track** | **Physical on track** | **Social-emotional on track** | **Learning on track** | **ECD index on track** |
| --- | --- | --- | --- | --- | --- |
|  | **OR/P (CI)** | **OR/P (CI)** | **OR/P (CI)** | **OR/P (CI)** | **OR/P (CI)** |
| Stunted and wasted | 0.63** [0.52 - 0.75] | 0.48 **[0.31 - 0.75] | 1.06 [0.90 - 1.23] | 0.62** [0.48 - 0.80] | 0.80** [0.68 - 0.95] |
| **Wealth quintile** | | | | | |
| First (lowest) (R) |  |  |  |  |  |
| Second | 1.67** [1.26 - 2.23] | 1.58 [0.85 - 2.96] | 1.15 [0.92 - 1.45] | 1.61* [1.11 - 2.33] | 1.38** [1.08 - 1.75] |
| Third | 2.47** [1.87 - 3.27] | 1.74 [0.91 - 3.31] | 1.15 [0.91 - 1.44] | 1.88** [1.28 - 2.77] | 1.78** [1.40 - 2.27] |
| Fourth | 3.00** [2.25 - 4.00] | 1.01 [0.56 - 1.82] | 0.90 [0.71 - 1.14] | 1.56* [1.05 - 2.32] | 1.53** [1.18 - 1.98] |
| Fifth (highest) | 5.45** [3.92 - 7.57] | 2.97* [1.08 - 8.20] | 1.34* [1.02 - 1.78] | 1.34 [0.84 - 2.12] | 2.35** [1.70 - 3.23] |
| **Caste/ethnicity** | | | | | |
| Brahmin/Chhetri (R) |  |  |  |  |  |
| Janajaties | 0.90 [0.73 - 1.11] | 1.24 [0.71 - 2.17] | 1.02 [0.85 - 1.22] | 0.76 [0.56 - 1.04] | 0.88 [0.72 - 1.07] |
| Dalit | 0.80 [0.61 - 1.04] | 0.68 [0.38 - 1.21] | 1.16 [0.92 - 1.45] | 0.91 [0.62 - 1.34] | 1.00 [0.78 - 1.27] |
| Other | 0.48** [0.33 - 0.70] | 0.33** [0.17 - 0.65] | 0.74* [0.54 - 1.00] | 0.83 [0.49 - 1.41] | 0.48** [0.35 - 0.66] |
| **Sex of head of household** | | | | | |
| Male (R) |  |  |  |  |  |
| Female | 1.56** [1.27 - 1.93] | 1.00 [0.60 - 1.67] | 1.17 [0.98 - 1.40] | 0.72* [0.54 - 0.96] | 1.32** [1.08 - 1.61] |
| **Household size** | | | | | |
| Less than five (R) |  |  |  |  |  |
| Five and above | 0.77** [0.64 - 0.94] | 0.78 [0.47 - 1.28] | 0.85 [0.72 - 1.00] | 0.95 [0.71 - 1.26] | 0.78** [0.65 - 0.94] |
| **Mother’s education level** | | | | | |
| None (R) |  |  |  |  |  |
| Basic (grade 1-8) | 2.12** [1.66 - 2.70] | 0.91 [0.54 - 1.53] | 1.07 [0.88 - 1.30] | 1.42* [1.03 - 1.97] | 1.52**[1.24 - 1.86] |
| Secondary (grade 9-12) | 3.74** [2.90 - 4.84] | 0.77 [0.43 - 1.37] | 1.26* [1.02 - 1.56] | 1.17 [0.82 - 1.65] | 2.10** [1.67 - 2.64] |
| Above secondary | 6.75** [4.32 - 10.55] | 2.11 [0.38 - 11.69] | 0.99 [0.69 - 1.43] | 1.57 [0.79 - 3.11] | 3.46** [2.16 - 5.55] |
| Child Sex | | | | | |
| Boy ® |  |  |  |  |  |
| Girl | 0.93 [0.78 - 1.11] | 1.22 [0.80 - 1.86] | 1.20* [1.03 - 1.39] | 0.97 [0.76 - 1.25] | 0.99 [0.84 - 1.16] |
| Child age in months | 1.11** [1.10 - 1.13] | 1.03* [1.00 - 1.07] | 1.01* [1.00 - 1.02] | 1.03** [1.01 - 1.05] | 1.07** [1.05 - 1.08] |

* p < 0.05; ** p < 0.01
